# Sequential extraction of compounds of interest from yeast biomass assisted by pulsed electric fields

**DOI:** 10.3389/fbioe.2023.1197710

**Published:** 2023-05-04

**Authors:** Alejandro Berzosa, Carlota Delso, Jorge Sanz, Cristina Sánchez-Gimeno, Javier Raso

**Affiliations:** Food Technology, Facultad de Veterinaria, Instituto Agroalimentario de Aragón-IA2, (Universidad de Zaragoza-CITA), Zaragoza, Spain

**Keywords:** yeast biomass, Pulsed electric fields, sequential extraction, glutathione, mannoproteins, β-glucans

## Abstract

One strategy to reduce cost and improve feasibility of waste-yeast biomass valorization is to obtain a spectrum of marketable products rather than just a single one. This study explores the potential of Pulsed Electric Fields (PEF) for the development of a cascade process designed to obtain several valuable products from *Saccharomyces cerevisiae* yeast biomass. Yeast biomass was treated by PEF, which affected the viability of 50%, 90%, and over 99% of *S. cerevisiae* cells, depending on treatment intensity. Electroporation caused by PEF allowed access to the cytoplasm of the yeast cell without causing total breakdown of the cell structure. This outcome was an essential prerequisite to be able to perform a sequential extraction of several value-added biomolecules from yeast cells located in the cytosol and in the cell wall. After incubating yeast biomass previously subjected to a PEF treatment that affected the viability of 90% of cells for 24 h, an extract with 114.91 ± 2.86, 7.08 ± 0.64, and 187.82 ± 3.75 mg/g dry weight of amino acids, glutathione, and protein, respectively, was obtained. In a second step, the extract rich in cytosol components was removed after 24 h of incubation and the remaining cell biomass was re-suspended with the aim of inducing cell wall autolysis processes triggered by the PEF treatment. After 11 days of incubation, a soluble extract containing mannoproteins and pellets rich in β-glucans were obtained. In conclusion, this study proved that electroporation triggered by PEF permitted the development of a cascade procedure designed to obtain a spectrum of valuable biomolecules from *S. cerevisiae* yeast biomass while reducing the generation of waste.

## 1 Introduction

Yeasts have long been involved in processes designed to obtain fermented foods such as wine, beer, and bread; today, the food industry also uses yeasts as alternative sources of high nutritional value proteins, conditioners, and flavoring agents (Dimopoulos et al., 2020). More recently, yeasts have started to be used for the production of specific molecules destined to be applied in the cosmetic and pharmaceutical industries, such as carotenoids, insulin, and recombinant proteins (Raskin and Clements, 1991; Mattanovich et al., 2012; Carlos Mata-Gómez et al., 2014). All these processes tend to generate considerable amounts of spent yeast. Yeast biomass is a raw material that contains several valuable compounds with a series of different potential uses (Li et al., 2004; Pérez-Serradilla and de Castro, 2008; Ferreira et al., 2010; Kritzinger et al., 2013; McClements et al., 2017). However, this type of waste has received little attention as a marketable commodity; what is more, its disposal often poses a series of economic and environmental challenges (Brito et al., 2007; Pérez-Bibbins et al., 2015).

Yeast biomass is mainly composed of proteins (35%–60% dry basis) of high biological value, because it contains all the essential amino acids (Bekatorou et al., 2006; Ganeva et al., 2020). Therefore, spent yeast is an excellent source that can be found in a widely available by-product, with excellent potential as an alternative source of high-quality proteins. On the other hand, yeasts synthesize a series of bioactive compounds that remain present in spent yeast. One of them is glutathione, a tripeptide which contains a reduced thiol group that confers antioxidant properties (Pastore et al., 2003). This compound, which protects cells from reactive oxygen species such as free radicals and peroxides, has found applications as an antioxidant in the food, cosmetic, and pharmaceutical industries (Li et al., 2004; Kritzinger et al., 2013).

The cell wall of yeast (15%–30% of its dry weight) is also a potential source of valuable compounds such as mannoproteins and β-glucans. Mannoproteins, which make up 35%–40% (w/w) of yeast cell wall dry weight, are highly glycosylated proteins located in the outermost layer of yeast cells, where they act as structural components (Quirós et al., 2012). The presence of mannoproteins in wine has been shown to enhance wine quality by improving stability and sensory properties (Pérez-Serradilla and de Castro, 2008). Various studies have demonstrated the emulsifying and stabilizing properties of mannoproteins by virtue of their amphipathic structure; they have also been associated with health-promoting properties of prebiotic ingredients, as they have been found to stimulate the growth of probiotic lactic acid bacteria (Ganan et al., 2012). On the other hand, the main component of the cell wall in yeast is β-glucans (50%–55% w/w), a glucose polymer with useful technological properties for the food industry as a thickener, as an emulsifying stabilizer, and by virtue of its water holding capacity (Zechner-krpan et al., 2009; Minh Tam et al., 2013). It has been also reported that ß-glucans may stimulate and improve the performance of the human immune system (Varelas et al., 2016; Bzducha-Wróbel et al., 2018).

Several studies exploring the valorization of spent yeast from brewing and winemaking processes have focused on obtaining a single valuable compound (Podpora B et al., 2015; Varelas et al., 2016; Vieira et al., 2016; Jacob et al., 2019). However, the valorization of by-products and the overall reduction of waste generation require the development of efficient, economical procedures. One strategy capable of increasing the efficiency of by-product valorization is that of obtaining a spectrum of marketable products from waste yeast biomass rather than just a single product. To achieve this objective, the implementation of Pulsed Electric Fields (PEF) technology as a pre-treatment applied to yeast biomass could serve as a useful first step in the design of a cascade processing sequence.

Pulsed Electric Fields is a technology that causes the loss of the selective permeability of the cytoplasmic membrane of cells by applying high-intensity electric fields (kV) for a very short period of time (µs). This phenomenon, called electroporation, is mainly associated with the formation of small pores in the cytoplasmic membrane that lead to uncontrolled molecular transport across it (Gehl, 2003; Álvarez et al., 2006; Mahnič-Kalamiza and Miklavčič, 2022). Electroporation facilitates the release of compounds from the interior of cells and has been used to recover a series of intracellular compounds from yeast, such as proteins, nucleic acids, and ionic substances (Ganeva et al., 2003; Liu et al., 2013; Dimopoulos et al., 2018). In addition, electroporation caused by PEF has been shown to trigger the autolysis process, which leads to the degradation of the cell wall by its own enzymes and to the concomitant release of mannoproteins (Martínez et al., 2016).

The revalorization of wastes and by-products generated in the food and biotechnology industry has attracted great attention in recent years due to its contribution to the bioeconomy strategy for sustainable growth. Although studies have been carried out for their revalorization, most of them were aimed at obtaining a single compound, although sometimes these by-products contain more than one valuable compound. In this sense, the aim of this study was to evaluate the potential of the use of PEF for the development of a cascade process that lead to the obtainment of several valuable products from *Saccharomyces cerevisiae* yeast biomass.

## 2 Materials and methods

### 2.1 Yeast strain and culture conditions

A commercial strain of *Saccharomyces cerevisiae* 3D viniferm (Agrovin, Ciudad Real, Spain), well known for its high mannoprotein production, was used in this investigation. Yeast cultivation was conducted in 1000 ml glass flask with 650 ml of Sabouraud-Dextrose broth (Oxoid, Basingtok, UK) under agitation at 25°C. Yeast growth was monitored by plate counting method in Potato-Dextrose-Agar (PDA) (Oxoid) after incubation of plates at 25°C for 48 h. The experiments were conducted on the yeast biomass containing cells in the stationary growth phase after 48 h of incubation.

### 2.2 PEF processing

#### 2.2.1 PEF treatment

Prior to treatment, fresh biomass of *S. cerevisiae* was centrifuged at 1593 x *g* for 10 min at 20°C and resuspended in citrate-phosphate McIlvane buffer of pH 7 and a conductivity of 2 ms/cm to a final concentration of 10^9^ CFU/ml (31.2 ± 0.8 g dry weight/L). This biomass was PEF-treated in a continuous flow chamber using a commercial PEF equipment (Vitave, Prague, Czech Republic) able to deliver pulses of up to 20 kV. This device can apply monopolar square waveform pulses of variable width (500 ns–100 µs) up to a maximum current intensity of 500 A and allowing to work up to 50 kHz.

A peristaltic pump (BVP, Ismatec, Wertheim, Germany) was used for pumping yeast biomass (5 L/h) through a titanium parallel electrode chamber of 0.4 cm gap, 3.0 cm length and 0.5 cm width. Square waveform monopolar pulses of 3 µs width were delivered at electric field strengths of 10, 15, and 20 kV/cm and frequencies lying between 39.8 and 159.3 Hz to reach total treatment times of 50–200 µs. These treatments corresponded to a total specific energy ranging from 11.5 to 207.9 kJ/kg of yeast suspension (31.2 ± 0.8 g dry weight/L). Actual voltage during treatments was measured by a high voltage probe (Tektronik, P6015A, Wilsonville, Oregon, United States) connected to an oscilloscope (Tektronik, TDS 220). Outlet temperatures were measured by a type K thermocouple inserted in the circuit (Ahlborn, Holzkirchen, Germany). After the treatments, suspensions were cooled down in less than 5 s to under 20°C in a heat exchanger located after the treatment chamber.

#### 2.2.2 Determination of the effect of PEF on viability of *S. cerevisiae*

After PEF treatments, serial dilutions of the yeast suspension in buffered peptone water (Oxoid) were plated on PDA to determine yeast viability after different treatment conditions. The number of viable cells, expressed as colony-forming units (CFU), corresponded to the number of colonies counted after 48 h of incubation at 25°C. Inactivation data were expressed as the % of inactivated cells calculated as the ratio between the initial number of cells (N_0_) and the number of viable cells after the treatment (N_t_).

### 2.3 Monitoring the release of compounds from *S. cerevisiae* treated by PEF

Once the effect of PEF on the viability of *S. cerevisiae* had been characterized, three PEF treatments, which affected approximately 50%, 90%, and over 99% of the yeast population, were selected to investigate the effect of electroporation on the release of a series of different compounds from the yeast biomass.

**FIGURE 1:**
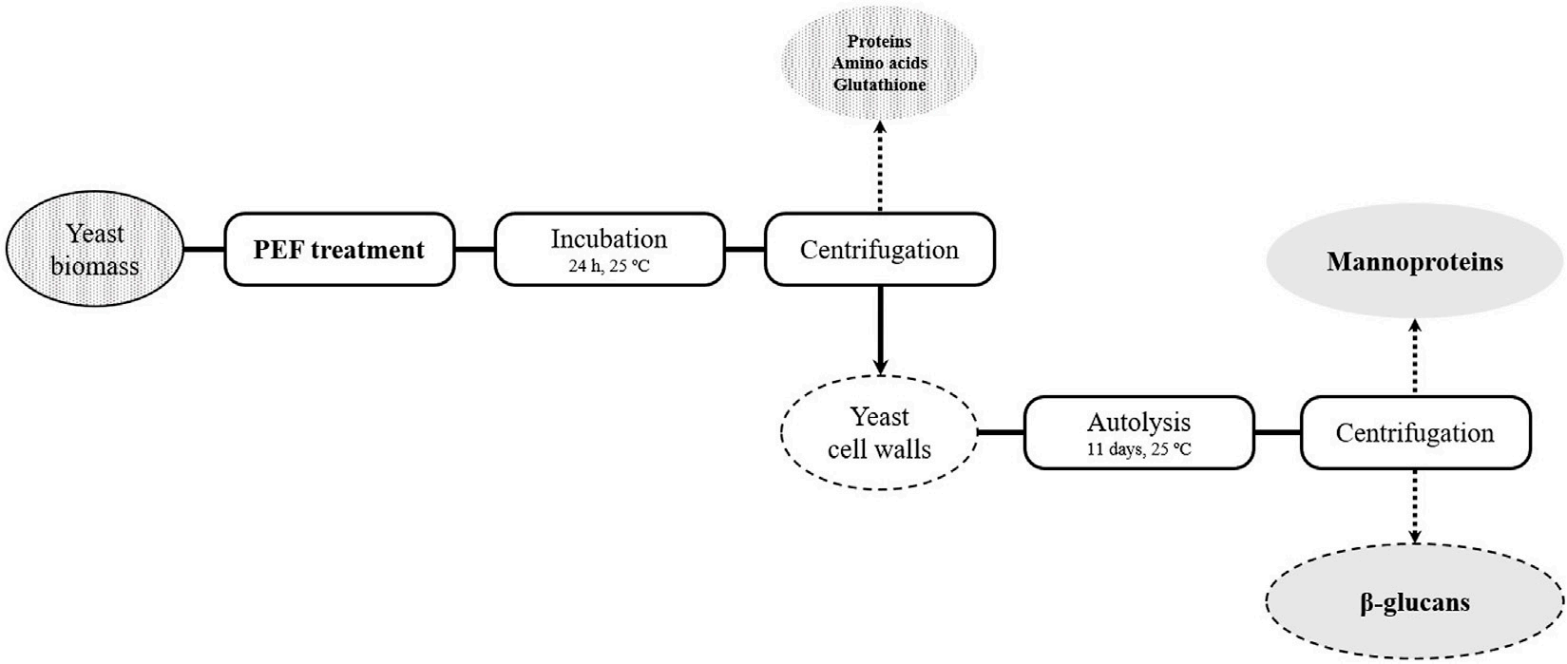
Outline of the cascade extraction process of *S. cerevisiae* compounds.

Following PEF treatments, yeast suspensions were incubated at 25°C for 24 h under agitation and compound release was monitored by spectrophotometric measurements (absorbance at 260 and 280 nm). After 24 h, suspensions were centrifuged at 1593 × g for 10 min in order to obtain the supernatant. Amounts of released protein, free α-amino nitrogen, and glutathione were determined. On the other hand, resultant pellets were resuspended in the same volume of McIlvane buffer pH 7 and incubated at 25°C for 11 days to monitor the release of mannoproteins from the cell wall during yeast autolysis. After incubation, a mannoprotein-rich supernatant and a β-glucans-rich pellet were obtained (Figure 1).

### 2.4 Bead mill treatment

To determine the total concentration of protein, free α-amino nitrogen, and glutathione present in the yeast cytoplasm, a bead milling treatment (Mini-Beadbeater-Plus; BioSpec, Bartlesville, United States) was applied. 1, 5 ml of the biomass described in Section 2.2.1 was placed in a 2 ml screw cap tube along with 0.5 mm diameter glass beads with a weight ratio of 1:5 (glass beads/yeast suspension). Mechanical disruption was monitored by microscopic observations (Eclipse E400, Nikkon, Tokyo, Japan). Fourteen (14) cycles of 70 s with 60 s of cooling in an ice-water bath between cycles were required for the destruction of over 90% of the cells. The suspensions were then centrifuged at 1593 × g for 10 min to obtain the supernatant in which the total concentration of compounds was analysed.

### 2.5 Dry cell weight determination

Dry weight of the samples was determined by removing water until a constant weight (30°C, 15 h) in a centrifugal concentrator (miVac DNA-23050-B00, Ipswich, England).

### 2.6 Analysis

#### 2.6.1 Spectrophotometric measurements

An aliquot of the suspensions was obtained; after centrifugation for 10 min at 3000 rpm in an Eppendorf AG centrifuge (Eppendorf, Hamburg, Germany), the absorbances at 260 and 280 nm in the supernatant were measured in order to monitor the release of intracellular material outside the cell (Aronsson et al., 2005). Results were presented as absorbance at 260 nm of the 1:100 dilution and absorbance at 280 nm of the 1:10 dilution in distilled water.

#### 2.6.2 Protein concentration

Protein extraction was determined by using the commercial Pierce BCA Protein Assay Kit (Thermo Fisher Scientific, Rockford, United States) based on the reduction of Cu^+2^ to Cu^+1^ by proteins in an alkaline medium (Biuret reaction) and colorimetric detection of cuprous cation (Cu^+1^) using a single reagent containing bicinchoninic acid (BCA). Briefly, 200 µl of the working reagent was added to 25 µl of sample (properly diluted in distilled water), shaken, and subsequently incubated at 37°C for 30 min. After the incubation period, absorbances were determined at 562 nm. A standard curve was prepared with albumin in a concentration range of 2.00 to 0.06 mg/ml. Results were expressed as mg of albumin equivalents per g of dry weight.

#### 2.6.3 Free α-amino nitrogen (FAN) concentration

The concentration of α-amino nitrogen (FAN) was quantified with the ninhydrin assay based on the procedure described by Dimopoulos et al. (2018), with modifications. The assay is based on the oxidized decarboxylation of alpha-amino acids caused by ninhydrin. The reduced ninhydrin reacts with unreduced ninhydrin, forming a blue complex with a strong absorbance at 570 nm. Briefly, 500 µl of extract (properly diluted in distilled water) was mixed with 250 µl of Ninhydrin Reagent (Sigma-Aldrich, Missouri, United States) and incubated for 15 min at 100°C. The suspensions were then cooled in an ice-water bath for 5 min, after which 1.25 ml of stop solution (0.2% KIO_3_ in 40% ethanol) was added to prevent further color development. The absorbance at 570 nm was read against a blank prepared with distilled water instead of extract. Results were expressed as mg of Glycine equivalents per g of dry weight.

#### 2.6.4 Determination of the concentration of reduced glutathione

The reduced form of glutathione (GSH) was determined by a colorimetric method with DTNB (5,5′-Dithiobis-(2-nitrobenzoic Acid)) (Thermo Fisher Scientific) following the procedure described by Ganeva et al. (2020), with some modifications. GSH reacts with DTNB, producing the chromophore TNB (5-thio-2-nitrobenzoic Acid) which has a maximum absorbance at 412 nm. Briefly, 960 µl of a phosphate buffered saline (PBS) pH 7.5 and 5.6 mM EDTA (Sigma-Aldrich) were added with 20 µl of a 0.4% DTNB solution prepared in the same buffer and another 20 µl of the sample. After incubation for 2–10 min at room temperature, absorbance was determined at 412 nm. Glutathione concentrations were determined from the standard curve made with reduced L-glutathione (Sigma-Aldrich) in a concentration range from 3.9 to 2000 μg/ml. Results were expressed as mg reduced L-glutathione per g of dry weight.

#### 2.6.5 Mannoprotein/mannose determination

Mannoproteins consist of mannose units bound to polypeptide chains. Mannoprotein release was indirectly determined by quantifying the concentration of mannose in the supernatant of the suspensions, after hydrolysis with sulfuric acid (final concentration of 1.5 M) at 100°C for 90 min and subsequent neutralization with NaOH (2 M). During this phase, the mannose chains constituting the mannoproteins are hydrolyzed to their monomeric form. Quantitative analysis of mannose concentration was carried out by an enzymatic method (D-Mannose, D-Fructose, D-Glucose Assay kit, Megazyme International, Wicklow, Ireland) (Dupin et al., 2000).

#### 2.6.6 Protease activity

Protease activity was measured using a commercial fluorescence assay kit (Protease Activity Assay Kit, Abcam, Cambridge, UK). The assay uses fluorescein isothiocyanate (FITC)-labeled casein as a general protease substrate. The fluorescein label of the FITC casein is highly quenched. After digestion by the proteases present in the sample, the FITC-Casein substrate is cleaved into smaller peptides which suppress the quenching of the fluorescent label. The fluorescence of the FITC-labeled peptide fragments is then measured at Ex/Em = 485/530 nm. A standard curve was realized with FITC Standard. Results were expressed as milliunits (mU) per ml of extract. One unit (U) is defined as the amount of protease that cleaves the substrate, to yield an amount of fluorescence equivalent to 1.0 μmol of unquenched FITC per minute at 25°C.

#### 2.6.7 β-glucosidase activity

β-glucosidase activity was tested using 4-nitrophenyl-β-D-glucopyranoside (pNPG) as substrate of the enzyme, resulting in the release of *p*-nitrophenol (pNPh), a pigmented substance measured spectrophotometrically at 400 nm. The assay was carried out following the procedure described by Hernández et al. (2003) with modifications. 1 ml of reaction mixture containing 5 mM of pNPG in McIlvane buffer pH 4.0 was mixed with 800 µl of extract (properly diluted in distilled water) and incubated at 50°C for 25 min. Subsequently, 2 ml of 1 M Na_2_CO_3_ was then added to stop the reaction. Absorbance at 400 nm was measured against reaction blank prepared by adding 2 ml of 1 M Na_2_CO_3_ to 800 µl of sample and then adding 1 ml of reaction mixture. β-glucosidase activity can be calculated using *p*-nitrophenol standard curve. Results were expressed as units (U) per ml of extract. One unit (U) is defined as the amount of β-glucosidase that hydrolyzes the substrate to generate an absorbance equivalent to 1 µmol of p-NPh per minute.

#### 2.6.8 β-glucan determination

Considering that β-D-glucan is a polymer of D-glucose units with β-1,3 and β-1,6 bonds, the concentration of β-D-glucan in the previously lyophilized pellets was determined using a commercial kit (Enzymatic Yeast Beta-Glucan Assay kit, Megazyme International) based on the quantification of glucose concentration by an enzymatic procedure after enzymatic hydrolysis. Briefly, 20 mg of previously lyophilized pellets were solubilized in 2 M potassium hydroxide with stirring in order to solubilize β-glucans; the pH was then adjusted to 4.0–4.5 with 1.2 M sodium acetate buffer. The solution was incubated with an enzyme preparation supplied by the kit for 16 h at 40°C, wherein the β-glucans were hydrolyzed to glucose monomers. After the incubation period and centrifugation, the glucose concentration in the supernatant was determined by an enzymatic procedure. Results were expressed as mg of β-glucans per g dry weight.

### 2.7 Statistical data analysis

Results represent the mean ± standard deviation of the mean of at least three replicates analyzed in duplicate. To establish significant differences among treatments, one-way analysis of variance (ANOVA) with a Tukey test was performed using Graph-Pad Software (San Diego, California, United States). Differences were considered significant at *p* < 0.05. The Pearson correlation coefficient was computed to assess the linear relationship between variables, with a 95% confidence interval, using Graph-Pad Software.

## 3 Results

### 3.1 Effect of PEF on the viability of *S. cerevisiae* cells

**FIGURE 2:**
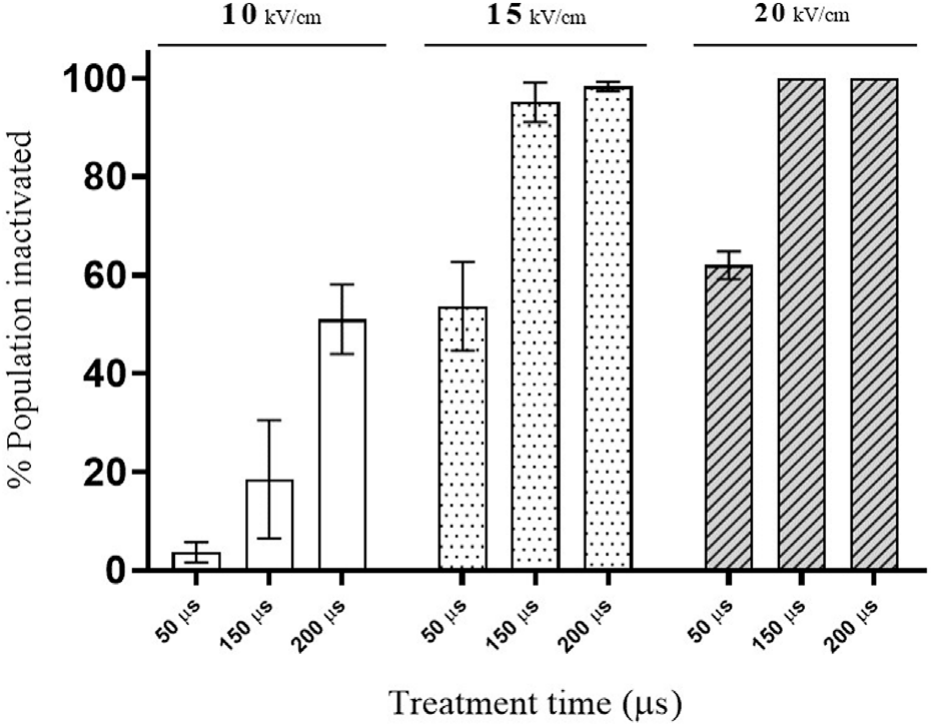
Effect of PEF treatments of different intensity on the viability of *S. cerevisiae* cells.

Figure 2 shows the influence of electric field strength and total treatment time, calculated by multiplying the total number of pulses applied by the pulse width, on % of *S. cerevisae* cells inactivated by PEF. At the lowest electric field investigated (10 kV/cm), the longest treatment (200 µs) caused a loss of viability in less of the 50% of the population. However, significant inactivation was detected at the lowest treatment time when the PEF treatments were applied at the two higher electric field strengths assayed (15 and 20 kV/cm). These results would indicate that the electric field threshold required for manifestation of electroporation of this strain of *S. cerevisiae* is around 10 kV/cm. Microbial inactivation by PEF is associated with the permanent permeabilization of the cytoplasmic membrane (electroporation), which leads to uncontrolled molecular transport across the membrane (Heinz et al., 2001; Álvarez et al., 2006; Mahnič-Kalamiza and Miklavčič, 2022). These data agree with those of other authors showing that electric fields higher than 10 kV/cm are required to achieve inactivation of several different strains of *S. cerevisiae* (Cserhalmi et al., 2002; Aronsson et al., 2005; Martínez et al., 2016).

Improving the extraction of intracellular compounds of interest requires the permanent electroporation of the cytoplasmatic membrane of yeast, similarly to total microbial inactivation. Consequently, in order to evaluate the usefulness of PEF for conceiving a cascade biorefinery designed to valorize yeast biomass with zero waste residue, treatment conditions affecting the viability of 50%, 90%, and over 99% of *S. cerevisiae* cells were selected. This could be achieved with treatments applied at both 15 kV/cm as well as 20 kV/cm. In view of commercially applying the technology on an industrial scale, lower electric field strength requirements are preferable. The treatments selected to obtain the desired inactivation rates were 15 kV/cm for 50 (PEF_1_), 150 (PEF_2_) and 200 µs (PEF_3_), which correspond to a total specific energy of 29.3, 87.7, and 117.1 kJ/kg of yeast suspension (31.2 ± 0.8 g dry weight/L) and outlet temperatures of 29.0, 42.9ºC and 49.9°C, respectively.

### 3.2 Release of UV-absorbing substances from cells treated by PEF

**FIGURE 3:**
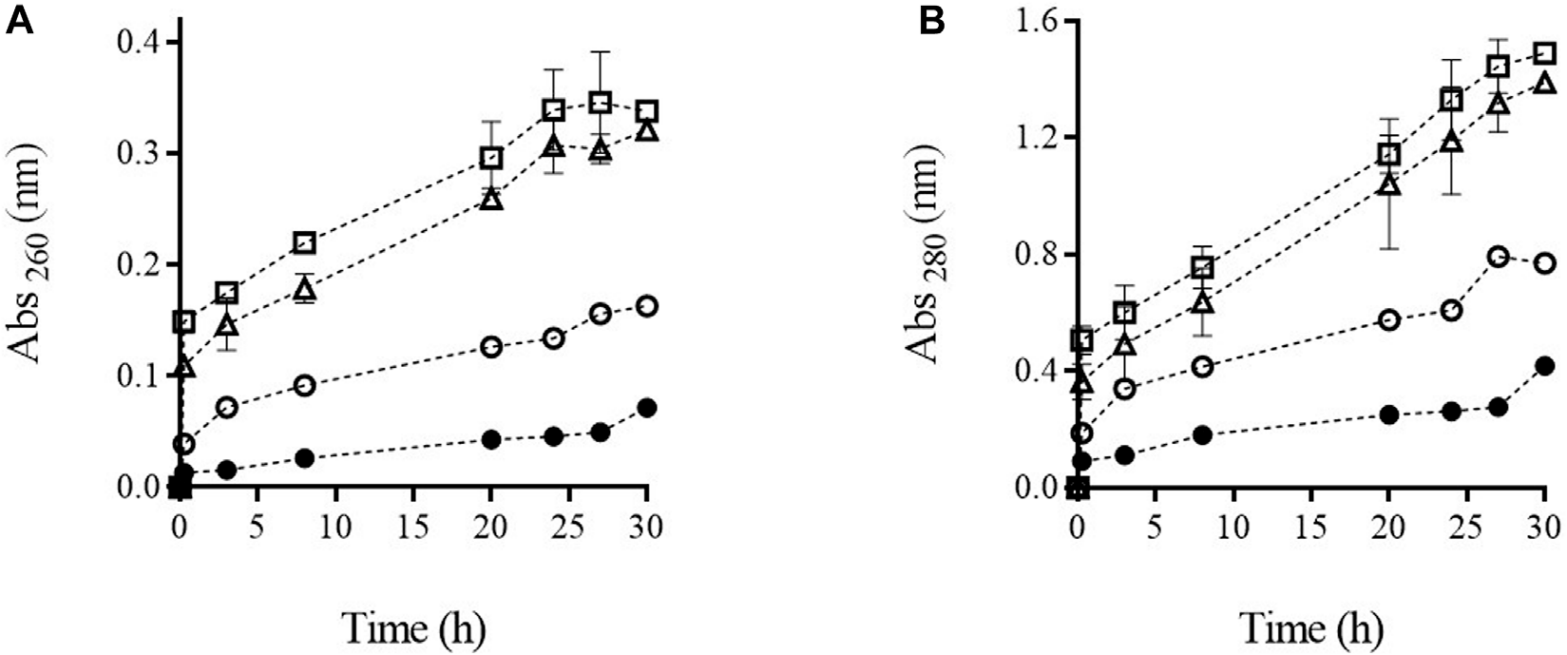
Absorbance at 260 nm **(A)** and 280 nm **(B)** of supernatant containing untreated (**●**) and PEF treated cells of *S. cerevisiae* cells at PEF_1_ (15 kV/cm 50 µs) (○), PEF_2_ (15 kV/cm 150 µs) (∆), and PEF_3_ (15 kV/cm 200 µs) (□) along the incubation time.

The presence of ultraviolet-absorbing material such as nucleic acid, proteins, and adenosine triphosphate (ATP) in the medium in which microbial cells are suspended is a common technique applied with the purpose of showing that electroporation has rendered the cytoplasmic membrane permeable to intracellular material that would otherwise be unable to cross it (Simpson et al., 1999; Cserhalmi et al., 2002; Martínez et al., 2016). Figure 3 illustrates the release of substances absorbing at 260 and 280 nm from the biomass of *S. cerevisiae* treated by PEF at different intensities. After the application of the two most intense PEF treatments (PEF_2_ and PEF_3_), an initial rapid increment of absorbance was observed immediately after the first 15 min of incubation. For example, in the biomass treated with the most intense PEF treatment (PEF_3_), the absorbance at 260 and 280 nm measured in the supernatant was more than half the maximum absorbance detected after prolonging incubation up to 30 h. This rapid increase in absorbance is assumed to be due to the leakage of low molecular weight compounds absorbing at 260 nm (ATP, nucleosides, and nucleotides) and at 280 nm (amino acids and small peptides that are free in the cytoplasm). Along the entire incubation period, the leakage of the intracellular substances that left the cytoplasm later was greater in the biomass that had been subjected to the most intense treatments. A comparable effect of the increment of intensity of the PEF treatment on the leakage of UV-absorbing substances at 280 nm was also observed by Yang et al. (2021) after having applied moderate pulsed electric fields (3–7 kV/cm) with a duration in the range of milliseconds. Our results showed that after prolonging the extraction to 30 h, no significant differences were detected in the absorbance at 260 and 280 nm in the supernatant containing biomass treated by PEF_2_ and PEF_3_. In the case of the supernatant containing the biomass in which 50% of the cells had been rendered non-viable by PEF, absorbance detected at 260 and 280 was 49% and 46% lower, respectively, after 30 h of incubation. Considering that the release of compounds from the untreated cells was almost negligible along incubation up to 30 h, these data indicate a positive correlation between the loss of viability of PEF-treated microbial cells and the UV-absorbing substances measured in the treatment medium, as has previously been reported (Aronsson et al., 2005). On the other hand, these results confirm that the main mechanism involved in microbial inactivation by PEF is the increment in permeability of the cytoplasmic membrane, caused by electroporation.

### 3.3 Extraction of amino acids, glutathione, and proteins from yeast biomass treated by PEF

**FIGURE 4:**
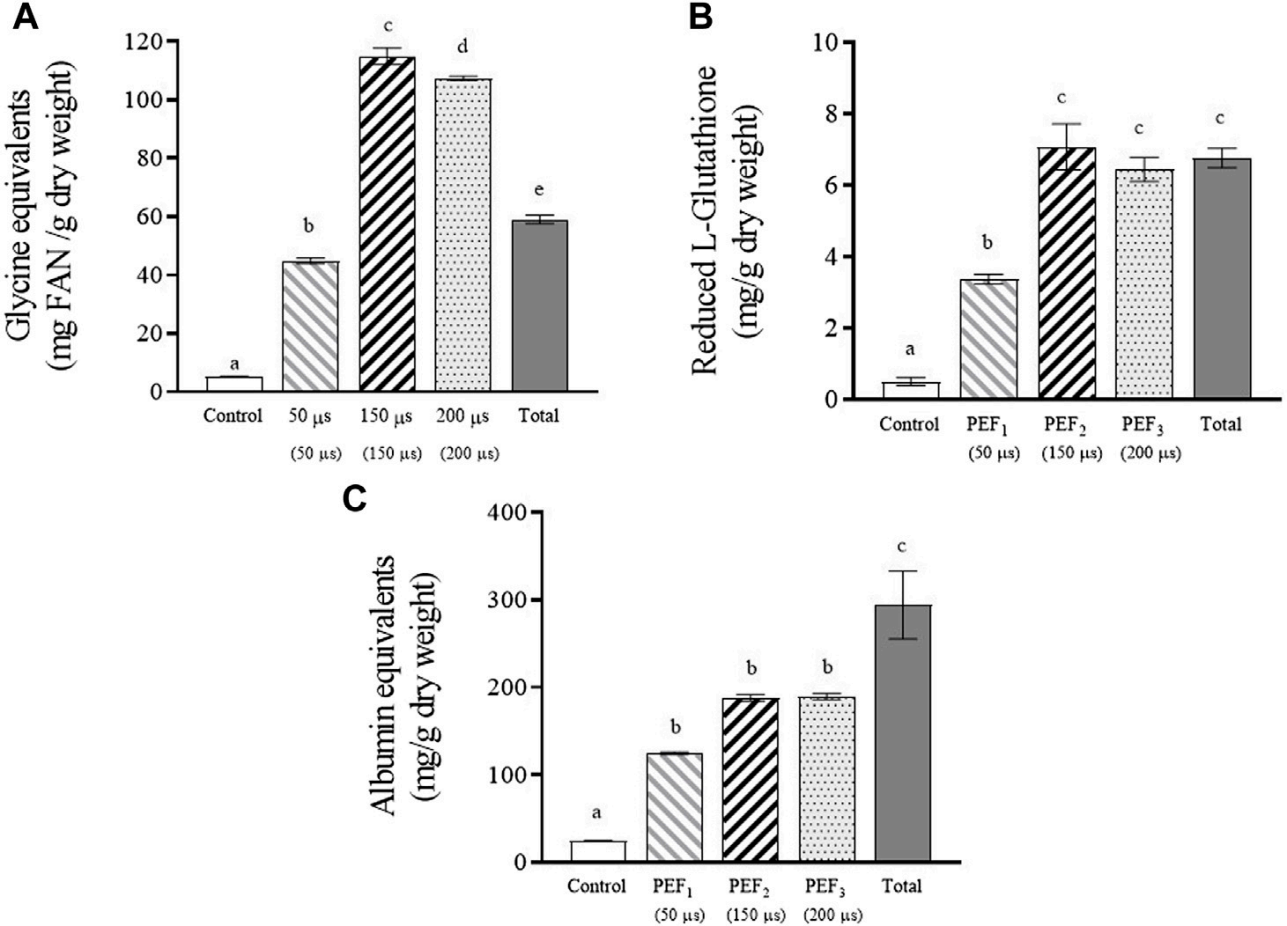
Free α-amino nitrogen **(A)**, reduced glutathione **(B)** and proteins **(C)** extracted after 24 h of incubation from untreated and PEF treated *S. cerevisiae* cells at 15 kV/cm for different times (50, 150, and 200 µs). Concentration of these compounds released from *S. cerevisiae* cells after beat milling (Total) is also shown. Different letters indicate significant differences (*p* ≤ 0.05).

The release of free α-amino nitrogen, glutathione, and proteins from *S. cerevisiae* cells subjected to the three assayed PEF treatments after 24 h of incubation are shown in Figure 4. The total content of these compounds in the supernatant of the yeast biomass after the complete destruction of the cells by bead milling is also shown.

Figure 4A shows the free α-amino nitrogen (FAN) content, which quantitatively expresses the total concentration of free amino acids and small peptides. The total amount of amino acids released from the yeast biomass after applying the least intense PEF treatment was slightly lower than the total content detected in the yeast biomass after bead milling. However, when the biomass had been treated at the highest intensities (PEF_2_ and PEF_3_), the amount of amino acids released after incubation was almost double. These differences in FAN content are comparable to those reported by other authors who observed 2.7 times more FAN in an autolysed yeast suspension than in one mechanically destroyed by bead milling (Jacob et al., 2019). This high amount of free amino acids compared to the amount detected after complete destruction of cells by bead milling could be due to hydrolysis of proteins in PEF-treated yeast cells catalyzed by proteases during the 24-h incubation period. It is well known that protease is a hydrolytic enzyme involved in yeast autolysis (Jones, 1991; Dimopoulos et al., 2021). Although shelf-degradation of yeast by its own enzymes during autolysis is a slow process lasting for more than 24 h, it has been demonstrated that electroporation triggers enzymatic activities in yeast, thereby accelerating autolysis (Martínez et al., 2016; Maza et al., 2020).

Figure 4B shows that after 24 h of incubation, the total amount of glutathione detected after bead milling was similar to the amounts released from the biomasses treated by PEF_2_ and PEF_3_ after 24 h of incubation. However, in the supernatant containing the cells treated by PEF_1_, the amount of extracted glutathione corresponded to half of that extracted with the most intense treatments. These results confirm that in terms of extracting low molecular weight compounds that may pass through the electroporated cytoplasmic membrane, there is a correspondence between the number of electroporated cells and the extraction yield.

Protein extraction (Figure 4C) from yeast biomass treated with the two most intense PEF treatments was less effective than amino acid and glutathione extraction. After 24 h of incubation, only 66% of the total amount of protein detected after beat milling was released in the supernatant containing the cells treated by PEF. This lower efficiency observed in the extraction of proteins could be related to the size of proteins. The passage of components located inside yeast cells to the extracellular medium requires the substance to pass through the cell wall in addition to the cytoplasmic membrane. Unlike molecules of lower molecular weight, certain macromolecules such as larger proteins might not be able to pass through the pores of the cytoplasmic membrane. But even if the cytoplasmic membrane was permeable to the largest proteins located within the cytoplasm, the cell wall could act as a barrier, preventing their passage to the supernatant. It was also interesting to observe that differences among the amount of proteins released from the biomass treated by PEF_1_ and the amount released from the biomass treated by the two most intense treatments were lower than for the other compounds of lower molecular weight. After 24 h of incubation, the protein detected in the extracellular medium containing yeast biomass with 50% electroporation was 65% of that extracted from biomass treated with PEF2 and PEF3. The relatively low amount of free amino acids released from the yeast biomass treated by PEF_1_ after 24 h of incubation (Figure 4A) seems to indicate that proteolysis did not occur or perhaps occurred to a lesser degree. Therefore, the amount of non-hydrolyzed proteins that can be released from yeast cells could explain the lower differences observed between the amount of protein release from the biomass treated by PEF_1_ compared with the amount released from biomass subjected to the two more intense PEF treatments.

On the other hand, adding the free amino acids content to the proteins content a similar value was obtained for the biomass treated by the most intense PEF treatments than for bead milling, as other authors have already reported (Jacob et al., 2019). These data show that by applying PEF and after 24 h of incubation, almost the 90% of the total free amino acids and proteins were released.

**FIGURE 5:**
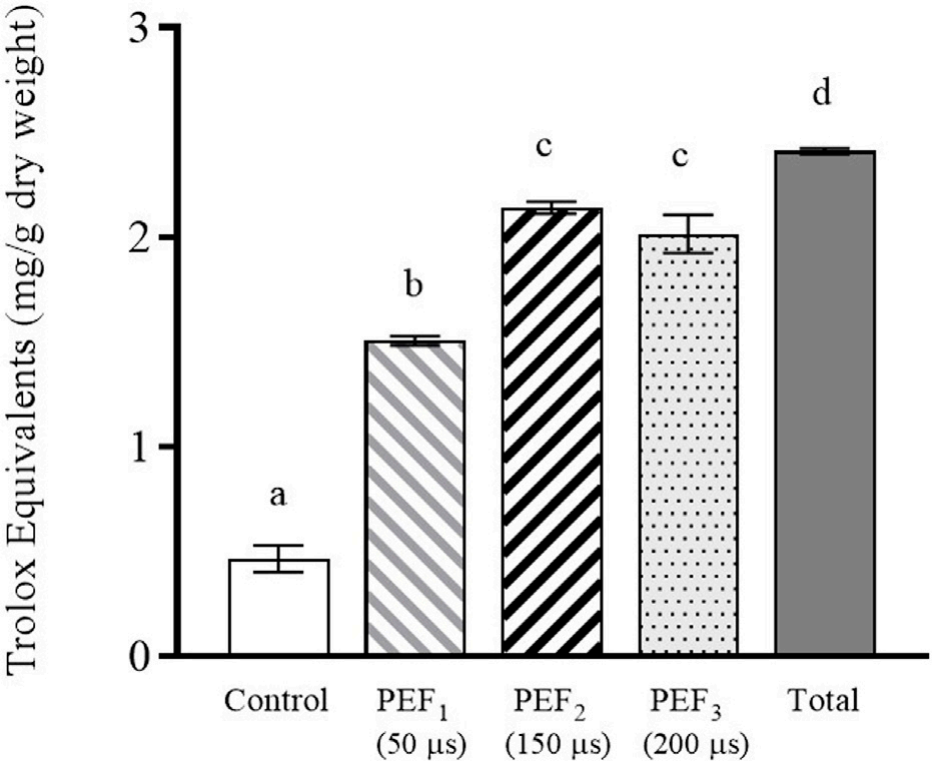
Antioxidant activity of the extract obtained after 24 h of incubation from untreated and PEF treated *S. cerevisiae* cells at 15 kV/cm for different times (50, 150, and 200 µs). Antioxidant activity of the *S. cerevisiae* cell extract after beat milling (Total) is also shown. Different letters indicate significant differences (*p* ≤ 0.05).

As mentioned above, glutathione is a molecule with a high antioxidant capacity. Therefore, the antioxidant capacity of the extracts was analyzed (Figure 5). Similarly to glutathione, the supernatant containing PEF_2_ and PEF_3_ treated cells showed the highest antioxidant capacity, namely 84%–89% of the total antioxidant activity detected in the supernatant containing cells after beat milling. A statistically significant correlation was observed between antioxidant capacity and glutathione content (Pearson correlation r = 0.97, *p* < 0.05), suggesting that antioxidant capacity may be influenced by the presence of glutathione, as previously reported by certain authors (Mirzaei et al., 2021). However, it is important to consider that other released molecules, such as proteins, peptides, and polyphenols, also exert an influence on antioxidant capacity. In this sense, we also observed a statistically significant correlation (Pearson r = 0.97, *p* < 0.05) between antioxidant capacity and the presence of proteins in the supernatant (Figure 4C). These results support the findings of other authors, who indicate that, in addition to glutathione, other molecules participate in the antioxidant capacity of yeast extracts (Bahut et al., 2020).

After incubating the yeast biomass subjected to the most intense PEF treatments for 24 h, we obtained an extract with 114.91 ± 2.86, 7.08 ± 0.64, and 187.82 ± 3.75 mg/g dry weight of amino acids, glutathione, and protein, respectively. As the objective of this study was to evaluate the use of PEF treatments to design a cascade biorefinery for the valorization of yeast biomass with zero residues, in a second step we removed the supernatant rich in amino acids, glutathione, and protein after 24 h of incubation, and re-suspended the remaining cell biomass in the same extraction medium with the purpose of obtaining valuable compounds located in the cell walls.

### 3.4 Obtaining mannoproteins and β-glucans from cell walls of PEF-treated biomass

**FIGURE 6:**
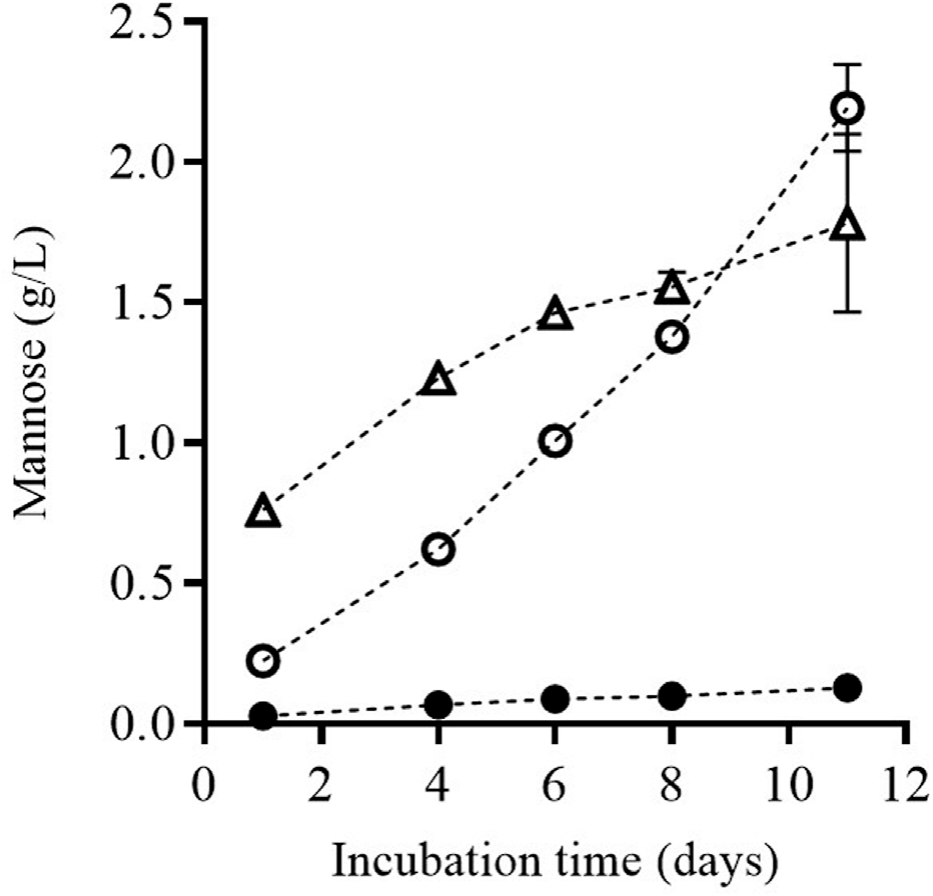
Release of mannose during incubation of untreated (**●**) and PEF treated *S. cerevisiae* cells at PEF_1_ (15 kV/cm for 50 µs) (○) and PEF_2_ (15 kV/cm for 150 µs) (∆), once the supernatant was removed after 24 h of incubation and the remaining cell biomass was re-suspended.

The release of mannoproteins from the cell wall to the surrounding media in which the yeasts are re-suspended requires the enzymatic degradation of the cell wall. Figure 6 shows the release of mannose from the yeast biomass cell wall along the incubation time, for the untreated biomass as well as for the biomass treated by PEF_1_ and PEF_2_. Determination of mannose released from the cell wall is a procedure used by several authors as an indicative value of mannoprotein release during yeast autolysis (Quirós et al., 2011; Martínez et al., 2016; Maza et al., 2020).

Along incubation time, the concentration of mannose in the medium in which the yeast cells were suspended was higher in the biomass that had been previously treated by PEF. Until Day 9 of incubation, the amount of released mannose was higher in the biomass subjected to the more intense PEF treatment (PEF_2_), but a longer incubation time did not increase it any further. However, mannose release increased by 23% in the biomass treated by PEF_1_. Consequently, after 11 days of incubation, the concentration of mannose was higher in the media containing the biomass treated by PEF_1_ than in the biomass treated by PEF_2_.

**FIGURE 7:**
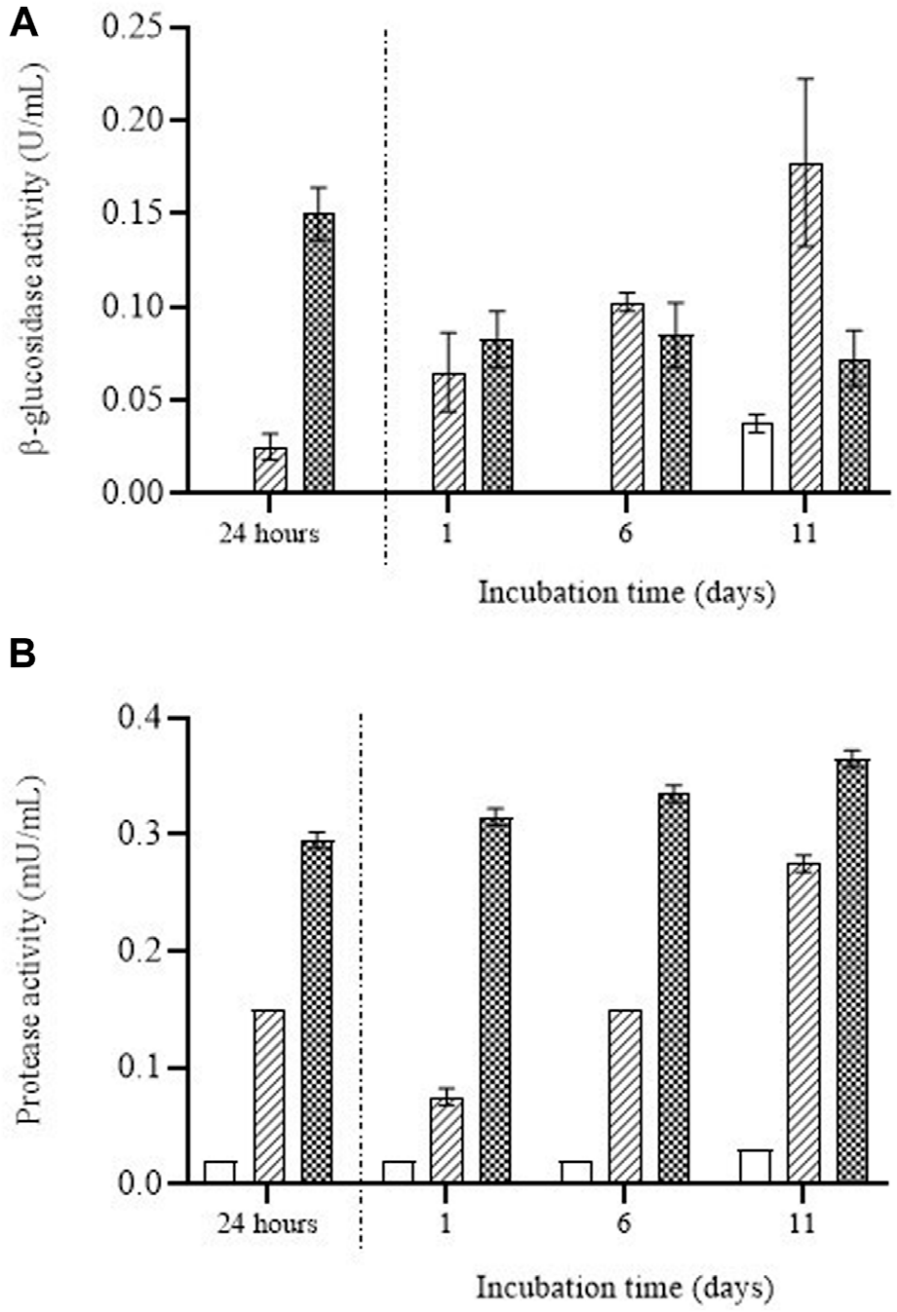
β-glucosidase activity **(A)** and protease activity **(B)** over the incubation time detected in the supernatant containing untreated (white bars) and PEF treated *S. cerevisiae* cells at 15 kV/cm for 50 µs (striped bars) and 150 µs (dotted bars) during the first 24 h of incubation and once the supernatant was removed after 24 h of incubation and the remaining cell biomass was re-suspended.

In order to assess the relationship between mannose release and the enzymatic degradation of the cell wall, we determined the activity of enzymes involved in the autolysis of yeast (protease and β-glucosidase) in the media containing the untreated and PEF-treated biomass (Figure 7). After the first 24 h of incubation, no hydrolytic activity of the two assayed enzymes was detected in the supernatant containing the untreated cells, but activity was detected in the supernatant containing the cells treated by PEF. Moreover, the activity was much greater in the biomass treated by PEF_2_, the most intense treatment. These results confirm that the natural degradation of cells by their own enzymes is a particularly slow process that requires the disorganization of membranous structures such as the cytoplasmic membrane and lysosome membranes. These findings also support observations made by other authors, who have shown that PEF contributes to the release of autolytic enzymes located in the lysosomes of the cytoplasm (Aguilar-Machado et al., 2020).

Figure 7 also shows that once the supernatant was removed after 24 h of incubation, enzymatic activity was still observed in the media in which the biomass had been re-suspended. In the case of the β-glucosidase enzyme (Figure 7A), after the first 24 h of incubation the enzymatic activity in the supernatant was greater in the cells treated by PEF_2_. After 1 day of incubation in the media in which the cells had been re-suspended, the enzymatic activity of β-glucosidase detected in the supernatant was similar for the cells treated by PEF_1_ and PEF_2_. But whereas the β-glucosidase activity of the medium containing PEF_1_-treated biomass increased along the incubation time, that activity remained constant for the PEF_2_-treated biomass. Similar behavior was observed in the case of protease (Figure 7B). A higher degree of protease activity was detected in the first 24 h of incubation for the biomass treated by PEF_2_ and after re-suspending the biomass. Although protease activity tended to increase with incubation in PEF_1_-treated biomass, it remained almost constant in the media containing the biomass subjected to the most intense PEF treatment (i.e., PEF_2_).

These results would indicate that after the first incubation period (24 h), β-glucosidase molecules and protease molecules were removed when the supernatant was separated from the biomass. However, a certain proportion of those autolytic enzymes remained in the cytoplasm of the cells, leading to cell wall degradation and subsequent release of mannoproteins into the extracellular medium. The higher degree of β-glucosidase activity persisting in the cell biomass treated at the lower electric field (PEF_1_) after removing the medium where the cells had been suspended for the first 24 h could explain why the amount of mannose was greater when the incubation time was extended.

**FIGURE 8:**
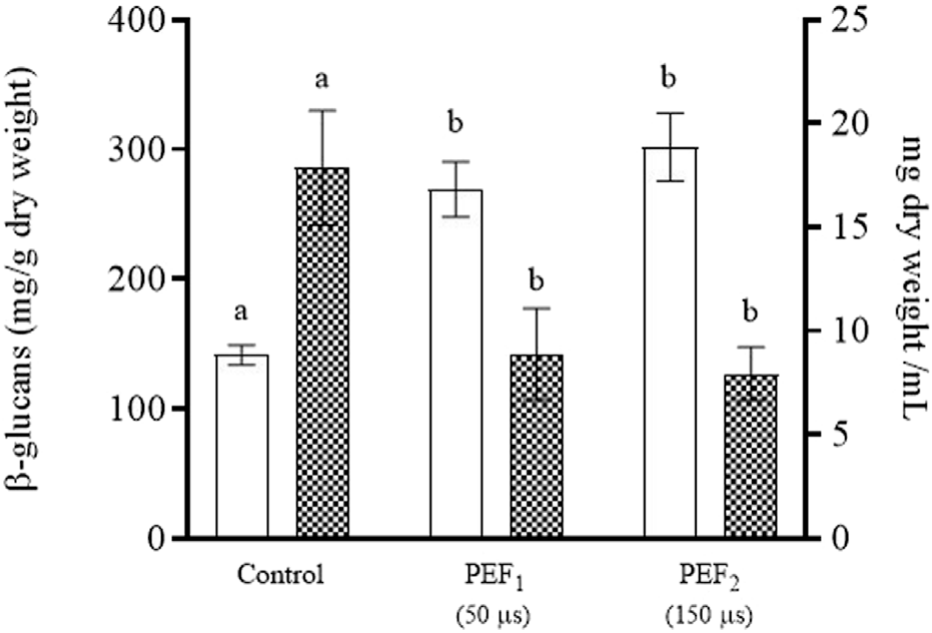
®-glucans (white bars) and dry weight (grid bars) of the pellet of untreated *S. cerevisiae* cells (control) and PEF-treated *S. cerevisiae* cells at 15 kV/cm for different times (50 and 150 µs) after 11 days of incubation. Different letters indicate significant differences (*p* ≤ 0.05).

In addition to mannoproteins, β-glucans are further valuable compounds located in the cell wall of yeast. Unlike mannoproteins, they are not soluble in water; thus, they were present in the precipitate obtained after separating the medium enriched in mannoproteins. Figure 8 shows the content of β-glucans and the dry weight of the pellets of the suspensions containing untreated yeast biomass as well as of those containing biomass treated at the two PEF intensities. The content of β-glucans expressed as mg of β-glucans per g of dry weight was approximately twofold in the biomass of the cells treated by PEF compared with the content in the biomass of untreated cells. However, the total amount of β-glucans was approximately the same in all sediments due to the fact that the dry weight of the yeast biomass treated by PEF amounted to around half the dry weight of the untreated biomass. This lower dry weight was due to the fact that a significant proportion of the yeast components had been removed in the two previous extraction steps, which had yielded a first extract rich in amino acids, glutathione and proteins, followed by a second extract rich in mannoproteins.

## 4 Discussion

This study aimed to evaluate the potential of PEF technology for the development of an efficient valorization process for yeast biomass generated by the food or biotechnological industries, with the aim of obtaining a range of valuable products instead of a single one. Its final purpose is to contribute to the objectives of the circular economy strategy by minimizing the waste generated by these industries.

Our research showed that the pores caused by PEF allowed access to the cytoplasm of the yeast cell without causing a total breakdown of the cell structure. This outcome was essential, because it opened up the possibility of performing a sequential extraction of the different value-added biomolecules from yeast cells located in the cytosol and cell wall. Mechanical rupture (using different procedures such as bead-milling, ultrasonication, or high-pressure homogenization) is the conventional procedure applied to provoke the release of biomolecules located in the cell cytoplasm (Liu et al., 2016; Jacob et al., 2019; Olivares-Galván et al., 2022). These procedures cause complete cell disruption, leading to a non-selective release of compounds and to micronization of cellular debris, thereby hampering the separation of valuable biomolecules in subsequent downstream processes. As an alternative, however, and as confirmed by our results, increasing the permeability of the cytoplasmic membrane by electroporation induces a selective and efficient release of compounds located in the cytoplasm of microbial cells such as yeast or microalgae without disintegrating the cell wall (Martínez et al., 2019). The data obtained in our study seem to indicate that the extraction efficiency of compounds located in the cytoplasm depends on the proportion of the population that was electroporated, rather than on the intensity of the treatment. A PEF treatment that electroporated 90% of the yeast biomass cells was more efficient in terms of extraction of amino acids, glutathione, and proteins than a treatment that electroporated 50% of the cells. However, a PEF treatment more intense than the one required to affect 90% of the population did not improve extraction efficiency.

Several studies have demonstrated the benefit of the application of a PEF treatment for the subsequent extraction of different compounds from yeast, such as proteins, carotenoids, glutathione, and recombinant proteins with antitumoral effect (Ganeva et al., 2015; 2020; Martínez et al., 2018; Aguilar-Machado et al., 2020; Guerrero-Ochoa et al., 2020). However, once those compounds of interest had been obtained, the rest of the cell, still containing valuable compounds such as β-glucans and mannoproteins, had been discarded. Therefore, with the aim of developing a sequential process designed to achieve a zero-residue multivalorization of yeast biomass, exhausted cells (after obtaining an extract rich in amino acids, glutathione, and proteins) were incubated again in an aqueous medium.

Release of mannoproteins into the medium in which the yeast biomass was re-suspended required enzymatic hydrolysis of the cell wall. Such a hydrolysis of the cell wall can be conducted with endogenous enzymes, mainly proteases and β-glucosidases. However, natural autolysis is a long-lasting process that requires the release of hydrolytic enzymes from lysosomes once cell death occurs. It has been recently demonstrated that cell electroporation by PEF triggers yeast autolysis. This effect has been associated with a rapid release of hydrolytic enzymes from plasmolyzed lysosomes as a consequence of the osmotic disequilibrium caused by water inlet to the cytoplasm of a cell of which the cytoplasmic membrane has been electroporated (Martínez et al., 2016). But it is also associated with the fact that the passage of the released enzymes through the cell wall is more efficient when the cytoplasm’s permeability has been increased by means of electroporation. Our results demonstrated that immediately after 24 h of incubation, β-glucosidase and protease activity were detected in the medium containing PEF-treated yeast biomass, thereby indicating that the treatment had promoted the release of hydrolytic enzymes. Although a proportion of the enzymes that had been released into the extracellular medium during the first 24 h of incubation were removed when the first extract was obtained, enzymatic activity was still detected in the media in which the yeast biomass was resuspended after incubation. The remaining enzymatic activity in the yeast biomass was sufficient to cause the release of mannoproteins from the cell wall and to obtain a second extract rich in that compound. Unlike the first extract in which the concentration of components was higher in the yeast biomass treated by the most intense PEF treatments, a greater amount of mannoproteins was obtained from the biomass treated with the less intense PEF treatment, when the extraction time was prolonged. These results could be explained by the fact that the amount of hydrolytic enzymes removed with the first extract was higher when the yeast biomass was treated with the most intense PEF treatment. Therefore, depending on the target compound one wishes to obtain, modulating the intensity of the PEF treatment applied to the yeast biomass at the beginning of the cascade extraction process can help to optimize the extraction of the compound in question. For example: when targeting mannoproteins as a compound to be released from the cell wall, a less intense treatment would be required in order to minimize the loss of hydrolytic enzymes through the first extract composed of compounds located in the cytoplasm.

The different degrees of water solubility of β-glucan and mannoproteins allowed for separation of these two compounds from the yeast cell wall. While the water-soluble mannoproteins passed on into the media in which the yeast biomass was suspended, insoluble β-glucans remained in the precipitated part after centrifugation.

Therefore, following the proposed approach, in this study, 3 fractions rich in different compounds of interest were obtained from the same yeast biomass, a first one after 24 h of incubation rich in amino acids, proteins and glutathione, a second one after 11 days of incubation rich in mannoproteins and a final pellet rich in β-glucans. In conclusion, this study proved that electroporation triggered by PEF allowed for the development of a cascade procedure designed to obtain a range of valuable biomolecules from *S. cerevisiae* yeast biomass while reducing the generation of waste. This biorefinery approach makes a positive contribution to the circular economy strategy and can be used to reduce and revalorize food industry waste, which generates voluminous amounts of yeast biomass. It can thus be applied in wineries and breweries, but also in the biotechnological industry with the aim of obtaining specific compounds from yeast. Further studies are required to more accurately determine optimal PEF treatments and extraction conditions depending on the main target compound to be obtained, as well as to evaluate the economic and environmental advantages of this approach.

## Data Availability

The raw data supporting the conclusion of this article will be made available by the authors, without undue reservation.

## References

[B1] Aguilar-Machado D. Delso C. Martinez J. M. Morales-Oyervides L. Montañez J. Raso J. (2020). Enzymatic processes triggered by PEF for astaxanthin extraction from xanthophyllomyces dendrorhous. Front. Bioeng. Biotechnol. 8, 857. 10.3389/fbioe.2020.00857 32903677 PMC7438825

[B2] Álvarez I. Condón S. Raso J. (2006). “Microbial inactivation by pulsed electric fields,” in Pulsed electric fields technology for the food industry: Fundamentals and applications. Editors Raso Javier V. Heinz (Boston, MA: Springer US), 97–129. 10.1007/978-0-387-31122-7_4

[B3] Aronsson K. Rönner U. Borch E. (2005). Inactivation of *Escherichia coli*, Listeria innocua and *Saccharomyces cerevisiae* in relation to membrane permeabilization and subsequent leakage of intracellular compounds due to pulsed electric field processing. Int. J. Food Microbiol. 99, 19–32. 10.1016/J.IJFOODMICRO.2004.07.012 15718026

[B4] Bahut F. Romanet R. Sieczkowski N. Schmitt-Kopplin P. Nikolantonaki M. Gougeon R. D. (2020). Antioxidant activity from inactivated yeast: Expanding knowledge beyond the glutathione-related oxidative stability of wine. Food Chem. 325, 126941. 10.1016/J.FOODCHEM.2020.126941 32387931

[B5] Bekatorou A. Psarianos C. Koutinas A. A. (2006). Production of food grade yeasts. Food Technol. Biotechnol. 44, 407–415.

[B6] Brito A. G. Peixoto J. Oliveira J. M. Oliveira J. A. Costa C. Nogueira R. (2007). “Brewery and winery wastewater treatment: Some focal points of design and operation,” in Utilization of by-products and treatment of waste in the food industry. Editors Oreopoulou V. Russ W. (New York: Springer). 10.1007/978-0-387-35766-9

[B7] Bzducha-Wróbel A. Błażejak S. Kieliszek M. Pobiega K. Falana K. Janowicz M. (2018). Modification of the cell wall structure of *Saccharomyces cerevisiae* strains during cultivation on waste potato juice water and glycerol towards biosynthesis of functional polysaccharides. J. Biotechnol. 281, 1–10. 10.1016/J.JBIOTEC.2018.06.305 29885339

[B8] Carlos Mata-Gómez L. César Montañez J. Méndez-Zavala A. Aguilar C. N. (2014). Biotechnological production of carotenoids by yeasts: An overview. Microb. Cell Fact. 13, 12. 10.1186/1475-2859-13-12 24443802 PMC3922794

[B9] Cserhalmi Z. Vidács I. Beczner J. Czukor B. (2002). Inactivation of *Saccharomyces cerevisiae* and Bacillus cereus by pulsed electric fields technology. Innovative Food Sci. Emerg. Technol. 3, 41–45. 10.1016/S1466-8564(01)00052-2

[B10] Dimopoulos G. Limnaios A. Aerakis E. Andreou V. Taoukis P. (2021). Effect of high pressure on the proteolytic activity and autolysis of yeast *Saccharomyces cerevisiae* . Innovative Food Sci. Emerg. Technol. 74, 102865. 10.1016/J.IFSET.2021.102865

[B11] Dimopoulos G. Stefanou N. Andreou V. Taoukis P. (2018). Effect of pulsed electric fields on the production of yeast extract by autolysis. Innovative Food Sci. Emerg. Technol. 48, 287–295. 10.1016/J.IFSET.2018.07.005

[B12] Dimopoulos G. Tsantes M. Taoukis P. (2020). Effect of high pressure homogenization on the production of yeast extract via autolysis and beta-glucan recovery. Innovative Food Sci. Emerg. Technol. 62, 102340. 10.1016/J.IFSET.2020.102340

[B13] Dupin I. V. S. McKinnon B. M. Ryan C. Boulay M. Markides A. J. Jones G. P. (2000). *Saccharomyces cerevisiae* mannoproteins that protect wine from protein haze: Their release during fermentation and lees contact and a proposal for their mechanism of action. J. Agric. Food Chem. 48, 3098–3105. 10.1021/jf0002443 10956076

[B14] Ferreira I. M. P. L. V. O. Pinho O. Vieira E. Tavarela J. G. (2010). Brewer’s Saccharomyces yeast biomass: Characteristics and potential applications. Trends Food Sci. Technol. 21, 77–84. 10.1016/j.tifs.2009.10.008

[B15] Ganan M. Carrascosa A. V. De Pascual-Teresa S. Martinez-Rodriguez A. J. (2012). Effect of mannoproteins on the growth, gastrointestinal viability, and adherence to caco-2 cells of lactic acid bacteria. J. Food Sci. 77, M176–M180. 10.1111/j.1750-3841.2011.02602.x 22384965

[B16] Ganeva V. Angelova B. Galutzov B. Goltsev V. Zhiponova M. (2020). Extraction of proteins and other intracellular bioactive compounds from baker’s yeasts by pulsed electric field treatment. Front. Bioeng. Biotechnol. 8, 552335. 10.3389/fbioe.2020.552335 33384987 PMC7770146

[B17] Ganeva V. Galutzov B. Teissié J. (2003). High yield electroextraction of proteins from yeast by a flow process. Anal. Biochem. 315, 77–84. 10.1016/S0003-2697(02)00699-1 12672414

[B18] Ganeva V. Stefanova D. Angelova B. Galutzov B. Velasco I. Arévalo-Rodríguez M. (2015). Electroinduced release of recombinant β-galactosidase from *Saccharomyces cerevisiae* . J. Biotechnol. 211, 12–19. 10.1016/j.jbiotec.2015.06.418 26142064

[B19] Gehl J. (2003). Electroporation: Theory and methods, perspectives for drug delivery, gene therapy and research. Acta Physiol. Scand. 177, 437–447. 10.1046/j.1365-201x.2003.01093.x 12648161

[B20] Guerrero-Ochoa P. Aguilar-Machado D. Ibáñez-Pérez R. Macías-León J. Hurtado-Guerrero R. Raso J. (2020). Production of a granulysin-based, tn-targeted cytolytic immunotoxin using pulsed electric field technology. Int. J. Mol. Sci. 21 (17), 6165. 10.3390/ijms21176165 32859066 PMC7503585

[B21] Heinz V. Alvarez I. Angersbach A. Knorr D. (2001). Preservation of liquid foods by high intensity pulsed electric fields—Basic concepts for process design. Trends Food Sci. Technol. 12, 103–111. 10.1016/S0924-2244(01)00064-4

[B22] Hernández L. F. Espinosa J. C. Fernández-González M. Briones A. (2003). $beta;-Glucosidase activity in a *Saccharomyces cerevisiae* wine strain. Int. J. Food Microbiol. 80, 171–176. 10.1016/S0168-1605(02)00149-6 12381403

[B23] Jacob F. F. Hutzler M. Methner F. J. (2019). Comparison of various industrially applicable disruption methods to produce yeast extract using spent yeast from top-fermenting beer production: Influence on amino acid and protein content. Eur. Food Res. Technol. 245, 95–109. 10.1007/s00217-018-3143-z

[B24] Jones E. W. (1991). Three proteolytic systems in the yeast *Saccharomyces cerevisiae* . J. Biol. Chem. 266, 7963–7966. 10.1016/s0021-9258(18)92922-4 2022624

[B25] Kritzinger E. C. Bauer F. F. Du Toit W. J. (2013). Role of glutathione in winemaking: A review. J. Agric. Food Chem. 61, 269–277. 10.1021/jf303665z 23240621

[B26] Li Y. Wei G. Chen J. (2004). Glutathione: A review on biotechnological production. Appl. Microbiol. Biotechnol. 66, 233–242. 10.1007/s00253-004-1751-y 15480625

[B27] Liu D. Ding L. Sun J. Boussetta N. Vorobiev E. (2016). Yeast cell disruption strategies for recovery of intracellular bio-active compounds — a review. Innovative Food Sci. Emerg. Technol. 36, 181–192. 10.1016/J.IFSET.2016.06.017

[B28] Liu D. Lebovka N. I. Vorobiev E. (2013). Impact of electric pulse treatment on selective extraction of intracellular compounds from *Saccharomyces cerevisiae* yeasts. Food Bioproc Tech. 6, 576–584. 10.1007/s11947-011-0703-7

[B29] Mahnič-Kalamiza S. Miklavčič D. (2022). “The phenomenon of electroporation,” in Pulsed electric fields technology for the food industry: Fundamentals and applications. Editors Raso Javier T. S. Heinz A. I. (Cham: Springer International Publishing), 107–141. 10.1007/978-3-030-70586-2_3

[B30] Martínez J. M. Cebrián G. Álvarez I. RasoJ. (2016). Release of mannoproteins during saccharomyces cerevisiae autolysis induced by pulsed electric field. Front. Microbiol. 7, 1435. 10.3389/fmicb.2016.01435 27672386 PMC5019107

[B31] Martínez J. M. Delso C. Angulo J. Álvarez I. Raso J. (2018). Pulsed electric field-assisted extraction of carotenoids from fresh biomass of Rhodotorula glutinis. Innovative Food Sci. Emerg. Technol. 47, 421–427. 10.1016/J.IFSET.2018.04.012

[B32] Martínez J. M. (2019). Innovative solutions based on the application of pulsed electric fields to improve the extraction of compounds of interest from microorganisms. Zaragoza, Spain: University of Zaragoza. [dissertation]. [Zaragoza].

[B33] Mattanovich D. Branduardi P. Dato L. Gasser B. Sauer M. Porro D. (2012). “Recombinant protein production in yeasts,” in Methods in molecular biology. Editor Lorence A. (Totowa, NJ: Humana Press), 329–358. 10.1007/978-1-61779-433-9_17 Recombinant Gene Expression 22160907

[B34] Maza M. A. Delso C. Álvarez I. Raso J. Martínez J. M. (2020). Effect of pulsed electric fields on mannoproteins release from *Saccharomyces cerevisiae* during the aging on lees of Caladoc red wine. LWT 118, 108788. 10.1016/j.lwt.2019.108788 30717010

[B35] McClements D. J. Bai L. Chung C. (2017). Recent advances in the utilization of natural emulsifiers to form and stabilize emulsions. Annu. Rev. Food Sci. Technol. 8, 205–236. 10.1146/annurev-food-030216-030154 28125353

[B36] Minh Tam T. Quoc Duy N. Phuoc Minh N. Thi Anh Dao D. (2013). Optimization of ßeta-glucan extraction from waste BREWER’S yeast SACCHAROMYCES cerevisiae using autolysis, enzyme, ultrasonic and combined enzyme – ultrasonic treatment. Am. J. Res. Commun. 1, 149–158.

[B37] Mirzaei M. Shavandi A. Mirdamadi S. Soleymanzadeh N. Motahari P. Mirdamadi N. (2021). Bioactive peptides from yeast: A comparative review on production methods, bioactivity, structure-function relationship, and stability. Trends Food Sci. Technol. 118, 297–315. 10.1016/J.TIFS.2021.10.008

[B38] Olivares-Galván S. Marina M. L. García M. C. (2022). Extraction of valuable compounds from brewing residues: Malt rootlets, spent hops, and spent yeast. Trends Food Sci. Technol. 127, 181–197. 10.1016/J.TIFS.2022.06.002

[B39] Pastore A. Federici G. Bertini E. Piemonte F. (2003). Analysis of glutathione: Implication in redox and detoxification. Clin. Chim. Acta 333, 19–39. 10.1016/S0009-8981(03)00200-6 12809732

[B40] Pérez-Bibbins B. Torrado-Agrasar A. Salgado J. M. Oliveira R. P. de S. Domínguez J. M. (2015). Potential of lees from wine, beer and cider manufacturing as a source of economic nutrients: An overview. Waste Manag. 40, 72–81. 10.1016/J.WASMAN.2015.03.009 25824282

[B41] Pérez-Serradilla J. A. de Castro M. D. L. (2008). Role of lees in wine production: A review. Food Chem. 111, 447–456. 10.1016/j.foodchem.2008.04.019 26047449

[B42] Podpora B. Świderski F. Sadowska A. Piotrowska A. Rakowska R. (2015). Spent brewer’s yeast autolysates as a new and valuable component of functional food and dietary supplements. J. Food Process Technol. 6. 10.4172/2157-7110.1000526

[B43] Quirós M. Gonzalez R. Morales P. (2012). A simple method for total quantification of mannoprotein content in real wine samples. Food Chem. 134, 1205–1210. 10.1016/j.foodchem.2012.02.168 23107749

[B44] Quirós M. Morales P. Pérez-Través L. Barcenilla J. M. Gonzalez R. (2011). A new methodology to determine cell wall mannoprotein content and release in wine yeasts. Food Chem. 125, 760–766. 10.1016/J.FOODCHEM.2010.08.066

[B45] Raskin P. Clements R. S. (1991). The use of human insulin derived from baker’s yeast by recombinant DNA technology. Clin. Ther. 13, 569–578. Available at: http://europepmc.org/abstract/MED/1799914 . 1799914

[B46] Simpson R. K. Whittington R. Earnshaw R. G. Russell N. J. (1999). Pulsed high electric field causes ‘all or nothing’ membrane damage in Listeria monocytogenes and *Salmonella typhimurium*, but membrane H+–ATPase is not a primary target. Int. J. Food Microbiol. 48, 1–10. 10.1016/S0168-1605(99)00022-7 10375130

[B47] Varelas V. Tataridis P. Liouni M. Nerantzis E. T. (2016). Valorization of winery spent yeast waste biomass as a new source for the production of β-glucan. Waste Biomass Valorization 7, 807–817. 10.1007/s12649-016-9530-4

[B48] Vieira E. Teixeira J. Ferreira I. M. P. L. V. O. (2016). Valorization of brewers’ spent grain and spent yeast through protein hydrolysates with antioxidant properties. Eur. Food Res. Technol. 242, 1975–1984. 10.1007/s00217-016-2696-y

[B49] Yang G. Wang R. Gao J. R. Niu D. Li J. Wen Q. H. (2021). The effect of moderate pulsed electric fields on autolysis of *Saccharomyces cerevisiae* and the amino acid content in autolysates. Int. J. Food Sci. Technol. 56, 441–451. 10.1111/ijfs.14659

[B50] Zechner-Krpan V. Petravić-Tominac V. Panjkota-Krbavčić I. Grba S. Berković K. (2009). Potential application of yeast β-glucans in food industry. Agric. Conspec. Sci. cus 74, 277–282.

